# Role of clinical pharmacist in ensuring safe medication practices for pediatric cardiac care in low- and middle-income countries

**DOI:** 10.1017/ash.2025.35

**Published:** 2025-02-26

**Authors:** Aniqa Batool, Rumana Sangi, Jibran Bin Yousuf, Abdul Sattar Shaikh, Muhammad Mohsin

**Affiliations:** 1 Clinical Pharmacist, National Institute of Cardiovascular Diseases, Karachi, Pakistan; 2 Pediatric Cardiologist, Aga Khan University Hospital, Karachi, Pakistan; 3 Head of Pharmacy Department, National Institute of Cardiovascular Diseases, Karachi, Pakistan; 4 Associate Professor, Pediatric Cardiologist, National Institute of Cardiovascular Diseases, Karachi, Pakistan; 5 Assistant Professor, Pediatric Electrophysiology, National Institute of Cardiovascular Diseases, Karachi, Pakistan

## Abstract

**Background and Objectives:**

Medication safety is critical for pediatric cardiac care, especially in low- and middle-income countries (LMICs), where limited resources contribute to high rates of medication errors. Studies in LMICs have shown that pharmacist interventions can reduce medication errors by up to 57% and proved that clinical pharmacists are essential for ensuring accurate and optimized medication use. This study aims to evaluate the role of clinical pharmacist interventions to rectify prescription errors in a pediatric cardiac care setting in Pakistan.

**Methods:**

This single-center retrospective study was conducted from January to August 2022 in a pediatric cardiac ward at the National Institute of Cardiovascular Diseases. Pediatric patients of all age groups and genders diagnosed with acquired, inherited, or congenital heart diseases were included. We reviewed patient files for any prescription changes made by the clinical pharmacist, based on a comprehensive review of the patient profile, treatment regimens, and laboratory results, to ensure safe and effective pharmacotherapy.

**Results:**

260 pharmacist interventions were observed among 2754 patients over eight months, demonstrating a significant role in mitigating medication errors. The interventions addressed wrong doses (126), incorrect frequencies (101), redundant coverage (23), and therapy duration errors (8). Antibiotics were the most frequent source of prescription errors, accounting for 81% of the interventions.

**Conclusion:**

Clinical pharmacists’ involvement in pediatric cardiac care significantly reduces medication errors in LMICs, as demonstrated by this study. These findings underscore the need to integrate clinical pharmacists into multidisciplinary teams to enhance medication safety and improve patient outcomes.

## Introduction

Globally, the prevalence of congenital heart disease (CHD) ranges from 4 to 50 per 1,000 live births, while in Pakistan, it is reported to be 3.4 per 1,000 live births. However, specific data on the prevalence of inherited cardiac diseases in Pakistani children remain limited. Furthermore, the prevalence of acquired heart conditions varies based on regional health factors.^1^ Pediatric cardiac disorders include congenital heart disease, arrhythmias, cardiomyopathies, valve disease, aneurysms, and cardiac inflammatory diseases (eg, pericarditis and myocarditis).^2–4^ Effective treatment includes a comprehensive, multidisciplinary approach, medical therapy, nutritional, developmental, and psychosocial support.^3,5^

Clinical pharmacist is important in optimizing pharmacotherapy in pediatric cardiac care, in developing countries.^6^ A systematic review of pharmacist-led interventions in LMICs revealed a 57% reduction in medication errors.^7^ Another study conducted in pediatric settings showed a decline in prescribing mistakes by up to 35%, particularly with antibiotics, antiepileptics, and cardiovascular drugs.^8^ These findings underscore the need for pharmacists to ensure the optimal use of medications. However, in Pakistan, this service remains limited due to a shortage of trained clinical pharmacists with expertise in managing medications for the pediatric cardiac population,^9^ especially in public-sector hospitals. In contrast, such services are predominantly available in private-sector hospitals.^10,11^

In this context, our study aims to address this gap by evaluating the role of clinical pharmacists in identifying and rectifying medication prescription errors in a pediatric cardiac care setting at a public hospital in Pakistan. The findings of this study will underscore the critical role of pharmacists in optimizing medication therapy in pediatric cardiac patients within public-sector settings in LMICs, particularly for this vulnerable population.

**Figure 1.**
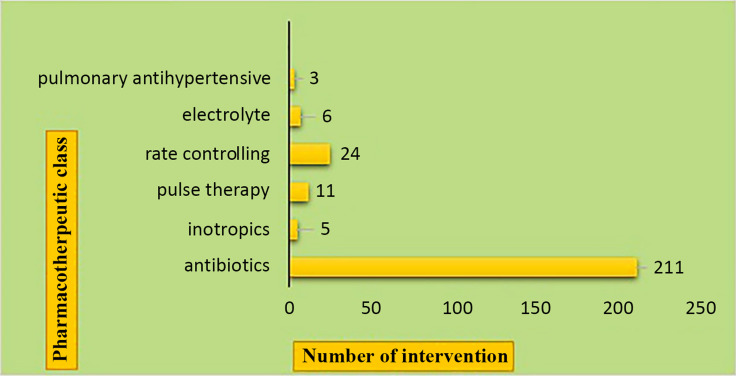
Prescription errors in the pharmacological categorical class identified and rectified by clinical pharmacist.

## Methods

**Study overview:** This is a retrospective clinical pharmacist experience on intervention over eight months (January to August 2022) on 2754 Pediatric cardiac patients admitted at the National Institute of Cardiovascular Disease (NICVD), Karachi, Pakistan. Probability consecutive sampling was employed to select the study participants from Pediatric cardiac patients admitted to the pediatric cardiac ward.

**Ethics approval and consent to participate:** This study was approved by the ethical review committee of the NICVD, Karachi, Pakistan with IRB-No. 39/2024. Due to the retrospective clinical experience nature of the study, ERC waived the patient’s consent.

**Procedure:** As a routine practice, clinical pharmacists were assigned to the pediatric cardiac ward. Their activities included reviewing patient profiles, confirming diagnoses, recording weight and height, evaluating treatment regimens, and interpreting laboratory results. Prescriptions were checked by calculating doses according to weight, frequency, and duration of therapy, and identifying the same pharmacotherapy medication regimen.^12^ When medication prescription errors were identified, the clinical pharmacist coordinated with the attending medical doctor to modify or change the prescription.^13^ The interventions were on the wrong dose, wrong frequency, incorrect duration of treatment, and unnecessary medication coverage.

**Data Collection:** We reviewed the patient file for any alteration or re-evaluation of prescriptions by the clinical pharmacist to the physician’s prescriptions based on a comprehensive review of the patient profile, assessment of treatment regimens, and interpretation of laboratory results to ensure safe and effective pharmacotherapy.

**Statistical Analysis:** Data were analyzed using the Statistical Package for Social Science Version 19. Data were summarized with the help of appropriate statistical measures such as mean ± standard deviation were reported for continuous variables and frequency (percentages %) was computed for the categorical variables.

## Results

### Overview

A clinical pharmacist observed 260 interventions in this study, with the number of interventions gradually declining over time, as shown in Table 1. Table 2 demonstrates the total number of interventions observed by the clinical pharmacist in rationalizing the medication therapy within eight months.

**Table 1.** Number of interventions per month

Month	Total admitted patients	Disease	CT-Angio	Cath	No. of Intervention
January	308	181	58	69	54
February	300	141	52	107	54
March	372	230	69	73	46
April	301	205	38	58	33
May	385	273	62	50	29
June	418	247	81	90	17
July	302	185	62	55	14
August	368	207	66	95	13

**Table 2.** Medication error prevention by pharmacist intervention in the pediatric cardiac ward

Drug	No. of interventions
Vancomycin	**34**
Ceftriaxone	**31**
cefotaxime	**26**
linezolid	**19**
Co-amoxiclav	**16**
Ceftazidime	**16**
Amikacin	**15**
Meropenem	**12**
Colomycin	**11**
Trimethoprim + sulfamethoxazole	**9**
Levofloxacin	**9**
Azithromycin	**7**
Ciprofloxacin	**3**
Metronidazole	**3**
Digoxin	**24**
Dobutamine	**3**
Dopamine	**2**
Methylprednisolone	**11**
Sildenafil	**2**
Bosentan	**1**
Potassium	**6**

### Types of interventions

The nature of clinical interventions included wrong dose: 126 interventions, wrong frequency: 101 interventions, same coverage: 23 interventions, days of therapy: 8 interventions, and both wrong dose and frequency: 2. The most frequent prescription errors were associated with the use of antibiotics (vancomycin, ceftriaxone, cefotaxime, ceftazidime, co-amoxiclav, linezolid, and amikacin), involving incorrect dose, frequency, or duration of therapy.

## Discussions

Globally, congenital heart disease (CHD) is the most common congenital anomaly associated with child mortality. The risk of medication errors heightens treatment complexity in CHD, necessitating stringent evaluation and management strategies.^14^ This study highlights the pivotal role of clinical pharmacists in ensuring safe medication use among pediatric cardiac patients in LMICs. The observed reduction in medication errors following pharmacist interventions highlights the need to integrate clinical pharmacy services into multidisciplinary teams to enhance medication safety and patient outcomes in pediatric cardiac care.

Our study corroborates with the findings, particularly in reducing antibiotic-related errors, which account for the highest proportion of prescribing errors in pediatric care globally. Given that antibiotics are often overprescribed or misused in LMICs, pharmacist-led interventions represent a critical step toward antimicrobial stewardship. For instance, research in Nigeria showed that pharmacist participation in multidisciplinary teams reduced inappropriate antibiotic use by 27% over one year^15^ whereas, in our study 10% reduction in inappropriate use of antibiotics.

Our findings are consistent with previous research by Elhabil et al., which identified 309 drug-related problems (DRPs) in 87 patients.^16^ In the present study, clinical pharmacists identified and resolved 260 DRPs among 2,754 pediatric cardiology patients over eight months. Similarly, a study conducted in Ethiopia reported that dosing errors were the most frequent issue, accounting for 37% of identified problems.^17^ In our study, dosing errors constituted the majority of interventions, with 48.5% of errors resolved by clinical pharmacists, highlighting their critical role in addressing medication safety concerns.

Studies have shown that pharmacist interventions in adult populations reduce medication errors, improve adherence, and enhance therapeutic outcomes,^18^ suggesting that similar benefits can extend to pediatric care. This study underscores the critical role of clinical pharmacists in optimizing medication use for vulnerable pediatric populations and highlights the need to integrate clinical pharmacy services into multidisciplinary teams to enhance medication safety and patient outcomes in pediatric cardiac care.

Despite these promising results, our study has several limitations. It is a single-center study, and the absence of a comparator group limits the generalizability of the findings. Additionally, the lack of full-time pharmacist involvement restricted comprehensive monitoring of adverse events, evaluation of hospitalization costs, food-drug and drug-drug interactions, review of discharge medication prescriptions, and assessment of emergency room visits. These constraints highlight the need for further research with robust study designs and expanded pharmacist involvement to fully elucidate the impact of clinical pharmacist interventions on patient outcomes.

## Conclusion

Clinical pharmacist intervention in pediatric cardiac care within LMICs, particularly in Pakistan, is an emerging field that holds significant promise for improving medication management and patient outcomes. While this study provides preliminary evidence of its potential benefits, it also underscores the critical shortage of trained clinical pharmacists in the region. This shortage may lead to suboptimal medication therapy and an increased risk of adverse events for pediatric cardiac patients. Although the results are promising, further research and a broader implementation of clinical pharmacy services are essential to confirm and expand upon these findings.

## Call to action for stakeholders to support and invest in pharmacist intervention programs

1. Analyzing the barriers to access to trained clinical pharmacists in pediatric cardiac care in LMICs and potential solutions to address this shortage.

2. Awareness regarding the importance of collaboration between healthcare providers, pharmacists, and other stakeholders to enhance patient safety and quality of care.

3. Examining successful pharmacist intervention programs implemented in other healthcare systems and how they could be adapted to benefit patients in LMICs.

4. Investigating the role of technology and telemedicine in bridging the gap between patients, healthcare providers, and pharmacists in resource-limited settings for improved medication therapy management.

## Data Availability

Data and materials will be available upon request.

## Patient and Public Involvement

No Patient or Public is involved.

## List of abbreviations

**LMIC Low and middle-income country**

**CHD Congenital heart defect**

**DRP drug-related problems**

**NICVD National institute of cardiovascular disease**
